# Human P2X7 Receptor Causes Cycle Arrest in RPMI-8226 Myeloma Cells to Alter the Interaction with Osteoblasts and Osteoclasts

**DOI:** 10.3390/cells9112341

**Published:** 2020-10-22

**Authors:** Ankita Agrawal, Lars S. Kruse, Annette J. Vangsted, Alison Gartland, Niklas R. Jørgensen

**Affiliations:** 1 Department of Clinical Biochemistry, Copenhagen University Hospital, Rigshospitalet, 2100 Copenhagen, Denmark; lars.kruse@leo-foundation.org (L.S.K.); niklas.rye.joergensen@regionh.dk (N.R.J.); 2 Department of Clinical Biochemistry, Research Institute Nordstjernevej 38, Rigshospitalet, 2600 Glostrup, Denmark; 3 Department of Hematology, Copenhagen University Hospital, Rigshospitalet, 2100 Copenhagen, Denmark; annette.juul.vangsted@regionh.dk; 4 The Mellanby Centre for Musculoskeletal Research, Department of Oncology and Metabolism, The University of Sheffield, Sheffield S10 2RX, UK; a.gartland@sheffield.ac.uk; 5 Department of Clinical Medicine, Faculty of Health and Medical Sciences, University of Copenhagen, 2100 Copenhagen, Denmark

**Keywords:** P2X7 receptor, myeloma, osteoblasts, osteoclasts

## Abstract

Multiple myeloma is a malignant expansion of plasma cells and aggressively affects bone health. We show that P2X7 receptor altered myeloma growth, which affects primary bone cells in vitro. Expression on six human myeloma cell lines confirmed the heterogeneity associated with P2X7 receptor. Pharmacology with 2′(3′)-O-(4-benzoylbenzoyl) adenosine 5′-triphosphate (BzATP) as agonist showed dose-dependent membranal pores on RPMI-8226 (*p* = 0.0027) and blockade with P2X7 receptor antagonists. Ca^2+^ influx with increasing doses of BzATP (*p* = 0.0040) was also inhibited with antagonists. Chronic P2X7 receptor activation reduced RPMI-8226 viability (*p* = 0.0208). No apoptosis or RPMI-8226 death was observed by annexin V/propidium iodide (PI) labeling and caspase-3 cleavage, respectively. However, bromodeoxyuridine (BrdU) labelling showed an accumulation of RPMI-8226 in the S phase of cell cycle progression (61.5%, *p* = 0.0114) with significant decline in G0/G1 (5.2%, *p* = 0.0086) and G2/M (23.5%, *p* = 0.0015) phases. As myeloma pathology depends on a positive and proximal interaction with bone, we show that P2X7 receptor on RPMI-8226 inhibited the myeloma-induced suppression on mineralization (*p* = 0.0286) and reversed the excessive osteoclastic resorption. Our results demonstrate a view of how myeloma cell growth is halted by P2X7 receptor and the consequences on myeloma–osteoblast and myeloma–osteoclast interaction in vitro.

## 1. Introduction

Multiple myeloma (MM) is a malignant growth of plasma cells and a unique neoplasia of hematologic origin. The latter is due to the bone-related complications in approximately 90% of MM patients owing to excessive bone destruction and insufficient bone formation [1]. Factors released during the bone remodeling favor myeloma growth and, in turn, myeloma cells contribute to cytokines that disrupt homeostatic bone turnover. Bisphosphonates are the mainstay treatment to preserve bone integrity, but morbidity and mortality remain significant [2]. Strategies counteracting the dependence of MM growth on the bone microenvironment have guided key discoveries including immunomodulators, surface antibodies, and immune checkpoint agents [3]. Whilst advances have extended survival in many patients, more avenues are needed to achieve a complete remission.

From an evolutionary standpoint, purines (adenosine-5′-triphosphate (ATP) and derivatives, ADP and adenosine) and pyrimidines (uridine-5′-triphosphate and uridine diphosphate) serve multiple purposes in living organisms. Other than a classical role in energy metabolism, they drive cellular proliferation, differentiation, and survival via specific cell-membrane receptors called purinergic P2 receptors (P2XR and P2YR) [4]. Basal purine and pyrimidine level and a steady-state P2 receptor activation sustain homeostasis in virtually all tissues [5]. In contrast, transient increases of ATP above steady-state levels are a signal of cellular stress. Subsequent defense responses involve purinergic signaling and layers of inflammatory pathways, such as those described in tumoral immunity [6,7]. Models of solid tumors conclusively show elevated ATP (extracellular ATP, eATP) in the tumor microenvironment (TME) thanks to innovative breakthroughs in methods of eATP detection [8,9,10]. These noninvasive measurements from pericellular spaces show eATP in levels of magnitude 10^3^–10^4^ times higher in the TME compared to healthy tissues (10^−9^–10^−6^ M) [10,11]. Thus, the importance of eATP has gathered most of the attention in the field of tumor biology. P2XR are more faithful to activation by ATP compared to P2YR and while P2XR subtypes 1–6 are engaged by lower levels of eATP (<10^−6^ M), P2XR subtype 7 (P2X7 receptor) requires higher eATP (>10^−6^ M) [12]. Features of P2X7 receptor involvement in cancer invasion/metastases in a variety of tumors is extensively reviewed [13,14,15].

In addition to the intrinsic properties of cancer cells and the biologics within TME, expression of P2X7 receptor on cancer cells or the host is also significant for malignant expansion [16,17]. The majority of evidence favors P2X7 receptor antagonism as a promising anti-cancer therapy [18]; however, P2X7 receptor-dependent apoptosis of epithelial cells can be a chemotherapeutic approach in cervical cancer [19]. Indirect effects where ATP triggers P2X7 receptor-induced immunogenicity and resolution of ATP signal such as with hydrolyzing enzymes (CD39 and CD73) generate a metabolic halo with contrasting immunosuppressive effects [6], blending purinergic signaling with tumor-immunology. Regulation of the immune system is intimately connected to the hematopoietic tissue—diseases affecting one will also affect the other. Understanding the participation of ATP and P2X7 receptor in hematological malignancies may therefore also lay the groundwork to combat tumor-induced immune suppression.

Direct effects of P2X7 receptor in myeloma biology are presented by in vitro studies [20,21]. However, this ATP/P2X7 receptor dependence is also reported in non-malignant peripheral blood and splenic B cells of human and murine origin, respectively [22], thus being non-specific to myeloma. The most common symptom of MM is bone pain and a direct function of P2X7 receptor in cancer-induced bone pain is unclear. P2X7 receptor antagonism in pre-clinical models are described [23,24] but despite accumulating evidence, the potential of the P2X7 receptor as an analgesic target remains unclear [25]. Discrepancies in findings are attributed to different treatment modalities, administration sites, potency, and kinetics of each drug candidate, and even then, any clinical relevance is limited by variations in the P2X7 receptor structure between rodents and humans.

In the present study, we address that P2X7 receptor alters myeloma growth and the downstream events following P2X7 receptor activation. Six human multiple myeloma cell lines (HMCLs)—RPMI-8226, U266, NCI-H929, OPM2, JJN3, and KMS12—were characterized. Expression of two transcript variants that alter P2X7 receptor function were determined by RT-PCR, and protein was visualized with two antibodies for specific but different epitopes on the P2X7 receptor. Functional features such as pore formation and ion channel, using pharmacological agents such as 2′(3′)-O-(4-benzoylbenzoyl) adenosine 5′-triphosphate (BzATP, more potent agonist than eATP) and antagonists with varying potency at the human P2X7 receptor were tested. We chose RPMI-8226 to identify events altered with P2X7 receptor activation. Lastly, we show that P2X7 receptor indirectly participated in the behavior of RPMI-8226 with bone cells using a novel triple-cell assay in vitro. Our results suggest that a functionally active P2X7 receptor is a target for intervention in the treatment of myeloma-induced bone disease.

## 2. Materials and Methods

Six bona fide HMCL (RPMI-8226, U266, NCI-H929, OPM2, JJN3, and KMS12) were maintained as per the description in the methods of the supplementary text. Cell culture reagent guidelines of ATCC and/or DSMG were followed unless stated otherwise. Primary osteoblasts and osteoclasts [26,27] were obtained from human volunteers after approvals from local ethics committees. Previously published P2X7 receptor primers [28] and inventoried TaqMan assays were used for gene expression. Total protein extracts (25 µg) were obtained from 3 subsequent passages and subjected to denaturing electrophoresis on Mini-PROTEAN Tris-Glycine eXtended gels on 3 different occasions. PVDF membranes (0.2 µm) were probed with P2X7R-ec (1.15 µg/mL) and P2X7R-Cter (0.6 µg/mL) and visualized by horseradish peroxidase (HRP)-conjugated anti-mouse IgG and anti-rabbit IgG, respectively. All experiments were performed when HMCL viability was determined as > 95% using trypan blue exclusion prior to seeding and/or starting treatments. Pore formation and Ca^2+^ influx were measured on live HMCL by YO-PRO-1 and Fluo-4 AM, respectively. Cell viability was determined by cell proliferation reagent (WST-1) and numbers by Quant-iT PicoGreen double stranded DNA. Apoptosis/necrosis (annexin V and propidium iodide (PI)) and cell cycle progression (pulse with 10 μM BrdU for 2 h) were analyzed on FACSVerse. A modified version of Boyden chamber with Corning HTS Transwell 96-well permeable supports (1.0 μm) was used for co-cultures (Figure S1). Detailed protocols are provided as supplementary text.

## 3. Results

### 3.1. P2X7 Receptor Was Expressed and Induced Calcium Influx on HMCL

**Figure 1:**
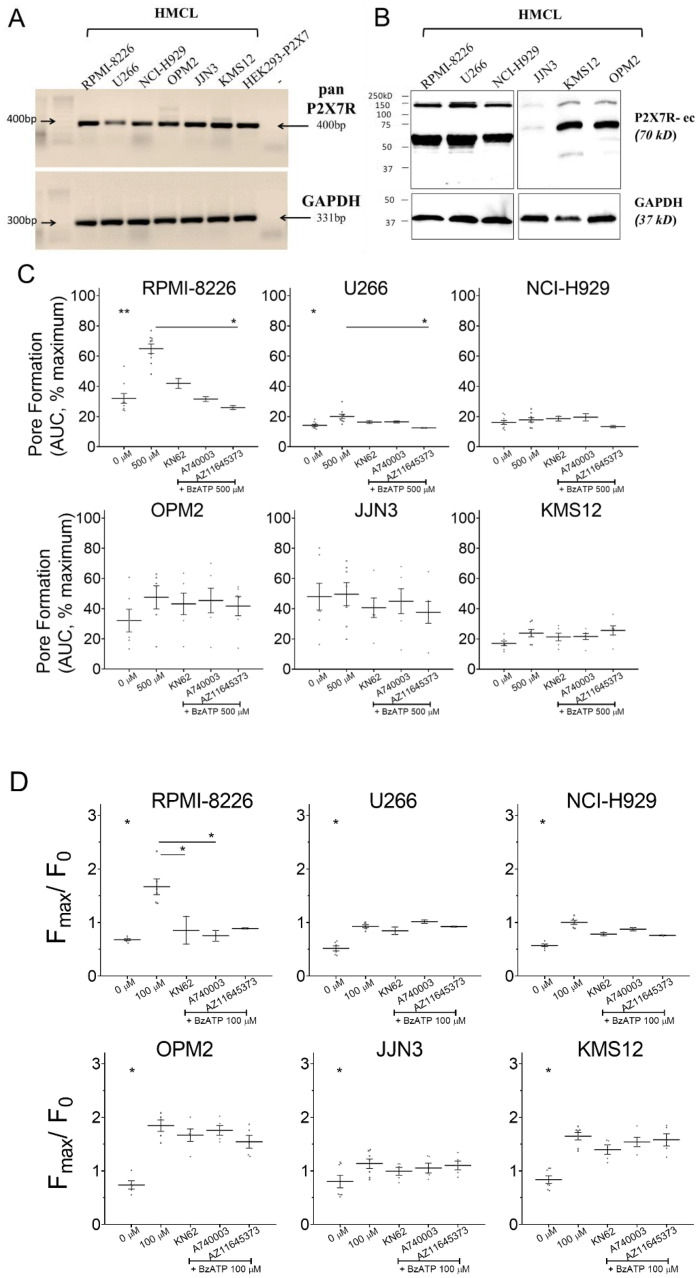
Expression and functionality of P2X7 receptor in RPMI-8226, U266, NCI-H929, OPM2, JJN3, and KMS12 human multiple myeloma cell lines (HMCLs). (**A**) Semi quantitative RT-PCR shows the P2X7 receptor mRNA expression in all HMCLs. Positive control was HEK293 cells stably transfected with P2X7 receptor, negative control (–) was a no template PCR reaction, and GAPDH expression corresponding to the same RT-PCR reaction was used as loading control. (**B**) Whole-cell lysates were immunoblotted with monoclonal antibody P2X7R-ec (raised against the extracellular domain of P2X7), and GAPDH (bottom panel) was the loading control. (**C**) Pore formation shown as area under the curve (AUC), with 500 µM 2′(3′)-O-(4-benzoylbenzoyl) adenosine 5′-triphosphate (BzATP) stimulus alone or in the presence of 10 µM P2X7 receptor antagonists (KN62, A740003, or AZ11645373), as indicated. Data are expressed as percentage of cell lysis (% maximum), achieved with injecting lysis reagent to each well at the end of measurement, and shows mean ± SEM of 6–9 independent experiments (*n*) with six replicate measurements per treatment (*n* = 2–4 with antagonists). * = *p* < 0.05, ** = *p* < 0.01, Kruskal–Wallis comparison with multiple corrections of each treatment to 500 µM BzATP. (**D**) Influx of Ca^2+^ evoked by 100 µM BzATP stimulus alone or in the presence of 1 µM P2X7 receptor antagonists (KN62, A740003, or AZ11645373), as indicated. Data are expressed as cytosolic [Ca^2+^] measured by peak fluorescence (F_max_) as a change from baseline (before stimulus, F_0_) and shows mean ± SEM of 5–7 independent experiments (*n*) with four replicate measurements per treatment (*n* = 2–5 with antagonists). * = *p* < 0.05, Kruskal–Wallis comparison with multiple corrections of each treatment to 100 µM BzATP.

Pan-P2X7 receptor primers that recognize all variants resulted in a 400bp size amplicon confirming a positive P2X7 receptor transcript (Figure 1A). Protein translation, using P2X7R-ec (recognizes epitopes 136–152 in the extracellular loop [29]) showed a ≈70kDa band in all HMCL (Figure 1B). An additional band at ≈150 kDa may have been a dimeric P2X7 receptor protein due to an incomplete subunit disassociation in our sample handling protocol. Next, P2X7 receptor pharmacology was determined by (a) pore-formation assay (YO-PRO-1 fluorescence with 500 µM BzATP stimulus) and (b) Ca^2+^ influx (Fluo-4 fluorescence by rise in cytosolic [Ca^2+^] with 100 µM BzATP). For pore formation, maximal YO-PRO-1 fluorescence (100%) was achieved by cell lysis at the end of each measurement. All values obtained with agonist stimulus (500 µM BzATP) are expressed as percentage of maximal. Pore dilation was significant in RPMI-8226 (64.9% versus 32% without agonist, *p* = 0.0067) and U266 (20.1% versus 14.2% without agonist, *p* = 0.0216) (Figure 1C). Pre-treatment with 10 µM AZ11645373, a P2X7 receptor-specific antagonist, prevented the BzATP-induced pore formation in both HMCL (25.9% in RPMI-8226, *p* = 0.0353 and 12.5% in U266, *p* = 0.0459). BzATP-induced pore-formation was absent all other HMCL (Figure 1C, Table 1). Activation of P2X7 receptor ion channel and influx of Ca^2+^ is expressed as peak fluorescence changed from baseline. Significant Ca^2+^ influx was seen in all six HMCL (Figure 1D, Table 1). Influx was highest in OPM2 (2.50-fold, *p* = 0.0009) followed by RPMI-8226 (2.46-fold, *p* = 0.0008), KMS12 (1.96-fold, *p* = 0.0002), U266 (1.77-fold, *p* = 0.0041), NCI-H929 (1.77-fold, *p* = 0.0001), and JJN3 (1.41-fold, *p* = 0.0413). Pharmacological blockade inhibited Ca^2+^ influx in RPMI-8226 but not in other HMCL (Table 1). Collectively we showed expression in all six HMCL but varying potency of agonist and a lacking inhibition with antagonists, reflecting differences in P2X7 receptor pharmacology between the HMCL.

### 3.2. P2X7 Receptor Expression, Function, and Signaling on RPMI-8226

Due to a variable pharmacology between the six HMCL, we utilized RPMI-8226 as a model to understand the potential of P2X7 receptor signaling in myeloma biology. RPMI-8226 expresses a pharmacologically sensitive P2X7 receptor and is also a common model of MM biology in vitro. Structurally, the human P2X7 receptor gene (*P2RX7)* is alternatively spliced into 10 transcript variants (www.ensembl.org). The full-length transcript designated as P2X7A has 13 exons and translates into 595 amino acid proteins. Nine variants are designated from P2X7B to P2X7J, but protein subunits are reported from P2X7B (P2RX7-203) and P2X7J (P2RX7-211) [30,31,32]. Evidence that heteromeric variant assembly alters the function usually associated with a homomeric P2X7A receptor [28], and variable stoichiometry of P2X7A:B subunits alters ligand sensitivity compared to a homomeric P2X7A receptor [30,33], provided a rationale to test these transcripts in RPMI-8226.

**Figure 2:**
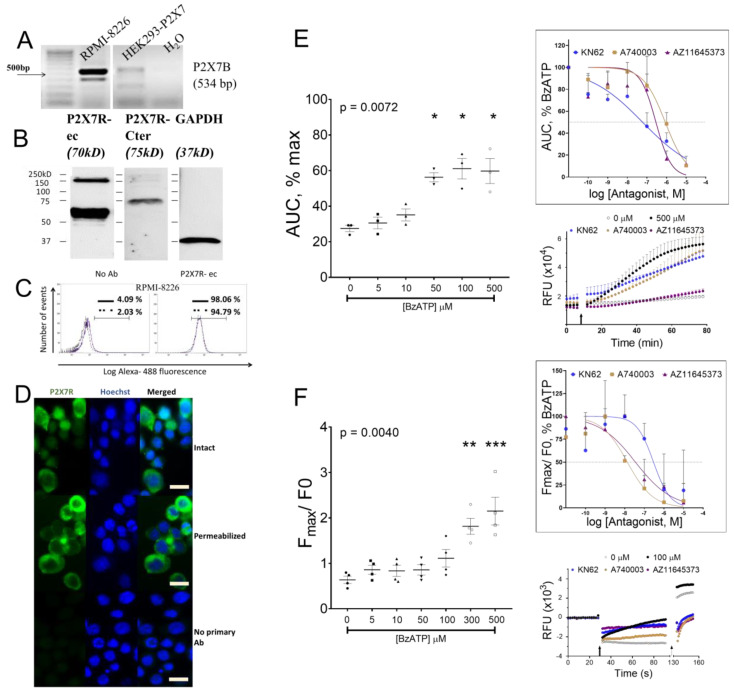
Characterization of P2X7 receptor in RPMI-8226. (**A**) Semi quantitative RT-PCR shows the transcript for truncated variant of P2X7 receptor compared to HEK293 cells stably transfected with P2X7 receptor used a positive control. (**B**) Whole-cell lysates were immunoblotted with antibody P2X7R-Cter (specific to the A isoform), showing a positive but lower intensity band compared to the P2X7R-ec (common to both A and B isoforms); GAPDH (right panel) was the loading control. (**C**) Histograms from flow cytometric analysis of intact (solid line) and permeabilized (dotted line) RPMI-8226 stained with monoclonal antibody P2X7R-ec or negative control (no Ab) with only secondary antibody to show percentage of positive cells (horizontal bar). (**D**) Microscopic images of RPMI-8226 stained with monoclonal antibody P2X7R-ec when intact (top), permeabilized (middle), and with no primary negative control (bottom). Hoescht was used to stain cell nuclei; scale bar 10 µm. (**E**) YO-PRO-1 uptake with increasing doses of BzATP shown as area under the curve (AUC) of maximum (cell lysis), and (inset) with 500 µM BzATP against increasing doses of antagonists (KN62, A740003, or AZ11645373). Injection trace of YO-PRO-1 uptake with BzATP (arrow marking the injection point) in the presence or absence of 1 µM KN62, 1 µM A740003, or 1 µM AZ11645373. Data shows mean ± SEM of three independent experiments (*n*) with six replicate wells per treatment (*n* = 2 with 6 replicate wells of each antagonist concentration) and * = *p* < 0.05, Kruskal–Wallis comparison with multiple corrections of each treatment to 0 µM BzATP. (**F**) Influx of Ca^2+^ evoked by increasing doses of BzATP, shown as peak fluorescence (Fmax) as a change from baseline (before stimulus, F0), and (inset) by 100 µM BzATP against increasing doses of antagonists (KN62, A740003, or AZ11645373). Injection trace of relative fluorescence (arrow marking the injection point; ionophore was used for full response shown at dotted arrow) in the presence or absence of 1 µM KN62, 1 µM A740003, or 1 µM AZ11645373. Data shows mean ± SEM of four independent experiments (*n*) with three replicate wells per treatment (*n* = 2 with duplicate wells of each antagonist concentration) and ** = *p* < 0.01, *** = *p* < 0.001, Kruskal–Wallis comparison with multiple corrections of each treatment to 0 µM BzATP.

A 534bp amplicon (Figure 2A) and positive immuno-reactivity to P2X7R-ec and P2X7R-Cter (C-terminal tail epitopes 576–595, APR-004) (Figure 2B) confirm the P2X7B variant. Quantitatively, larger immuno-reactivity to P2X7R-ec indicate subunits with the extracellular loop in comparison to the C-terminal tail (P2X7A). Flow cytometry after gating to exclude cell debris, doublets, and Hoechst positivity showed the majority of RPMI-8226 with fluorescent P2X7R-ec (98%, solid line, Figure 2C). Membrane permeabilization using 0.1% triton-X slightly reduced the positive population (94.8%, dotted line, Figure 2C). Median fluorescence intensity (MFI), an index of receptor number per cell, was reduced with permeabilization of RPMI-8226 (MFI = 561 versus 591 on intact cells), indicating a decline in P2X7 receptor surface density. This was confirmed by microscopic visualization with positive reactivity to P2X7R-ec on the majority of RPMI-8226 compared to a lower intensity, peri-nuclear staining pattern with triton-X (Figure 2D).

To confirm our earlier observations of a functional P2X7 receptor on RPMI-8226, we performed a detailed pharmacological analysis including a dose-dependent agonist response and antagonism with P2X7 receptor antagonists. BzATP at doses ≥ 50 µM induced significant pore formation (*p* = 0.0072), which was prevented by pretreatment with three different P2X7 receptor antagonists (Figure 2E). IC_50_ values, calculated by non-linear curve with robust fit, were 0.05 µM, 0.85 µM, and 0.29 µM for KN62, A740003, and AZ11645373, respectively (Figure 2E, inset). Similarly, BzATP-induced influx of Ca^2+^ (*p* = 0.0040) was inhibited with KN62, A740003, and AZ11645373 pretreatment (Figure 2F). IC_50_ values were 0.29 µM, 0.01 µM, and 0.03 µM for KN62, A740003, and AZ11645373, respectively (Figure 2F, inset). Collectively, our results show the potency of BzATP and P2X7 receptor antagonists in RPMI-8226.

### 3.3. P2X7 Receptor Restricted Number of Viable RPMI-8226 by Cell Cycle Arrest and Not Apoptosis

**Figure 3:**
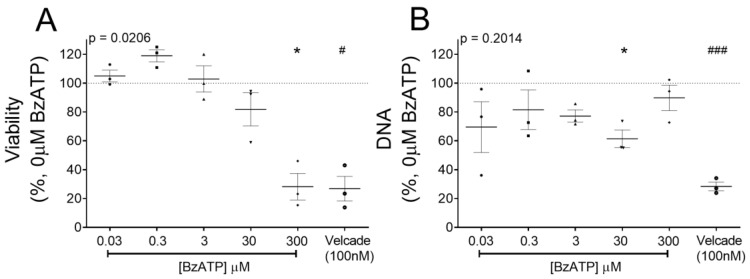

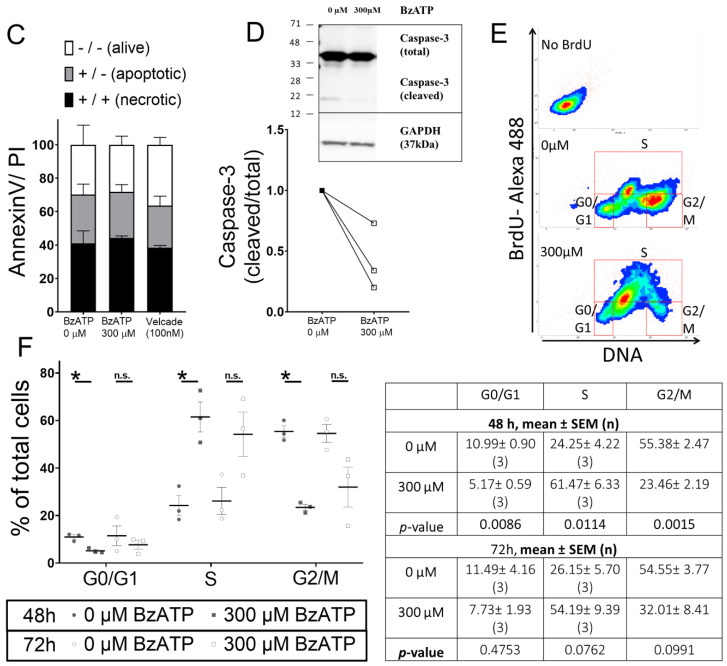
P2X7 receptor induced cell cycle arrest but not apoptosis in RPMI-8226. (**A**) Viability as a measure of metabolically active and (**B**) DNA content (pg/well) of RPMI-8226 cells after 48 h with BzATP is plotted as a percentage of control (0 µM BzATP, dotted line). Velcade (100 nM), an anti-myeloma agent, was used as a positive control. Data shows mean ± SEM of three independent experiments; Kruskal–Wallis comparison to show * = *p* < 0.05 multiple corrections of each treatment to 0 µM BzATP (# = *p* < 0.05, ### = *p* < 0.001 Mann–Whitney test for 0 µM BzATP vs. Velcade). (**C**) Flow cytometry to assess the percentage of alive (annexin V-/PI-), apoptotic (annexin V+/PI-), and necrotic (annexin V+/PI+) cells after 48 h with 300 µM BzATP. Data shows mean ± SEM of four independent experiments. (**D**) Western blot analysis to quantify caspase-3-cleaved protein (17 kDa) as a ratio of total protein (35 kDa) after 48 h with 300 µM BzATP; GAPDH was probed as a loading control (*n* = 3). (**E**) Scatter diagrams representing the populations in G0/G1, S, and G2/M phases of the cell cycle as determined by flow cytometry and (**F**) analysis following a 2-h BrdU pulse showing the percentage of cells in each cell cycle phase after 48 and 72 h treatment with 300 µM BzATP. Data shows mean ± SEM of three independent experiments, where * = *p* < 0.05 by Mann–Whitney test compared to 0 µM BzATP in the corresponding cell cycle phase.

Sustained P2X7 receptor activation induces RPMI- 8226 cell death [20]. Metabolic activity, as a measure of viable RPMI-8226, was measured after chronic P2X7 receptor activation (300 µM BzATP for 2 days). Viability was significantly reduced (28.2%, *p* = 0.0388) (Figure 3A) and comparable to treatment with an extensively used anti-myeloma agent, Velcade (26.8% viability, *p* = 0.0135). Curiously, 300 μM BzATP did not reduce dsDNA content, a measure of cell numbers (89.8% dsDNA versus control) (Figure 3B), suggesting that the decline was independent of cell death. To determine whether apoptosis is responsible for a lower percentage of viable cells, we assessed presentation of plasma membrane protein annexin V (a marker of apoptosis), together with propidium iodide (PI; permeant to dead cells). Flow cytometry showed no significant differences in the percentage of viable (annexin V−PI−), apoptotic (annexin V+/PI−), and necrotic (annexin V+/PI+) RPMI-8226 after 2 days of BzATP treatment (Figure 3C). However, RPMI-8226 demonstrated high variability between the four experimental replicates and even treatment with Velcade, a known mechanism via cell apoptosis, failed to induce apoptosis in our study. Therefore, we immunoblotted for caspase-3, a P2X7 receptor-dependent caspase, for apoptotic activity and cell death. BzATP reduced caspase-3 cleavage (17 kDa) (0.42-fold, Figure 3D), reflecting a reduction in apoptosis of RPMI-8226.

Myeloma cells have several mechanisms that allow them to evade apoptosis and contribute to their survival. One such phenomenon is an evolved cell-cycle behavior, and while disruptors of cell cycle progression are not fully understood, patient relapses are attributed to the cycle re-entry and subsequent expansion of the malignant clone. Therefore, we assessed the cell-cycle behavior of RPMI-8226 using BrdU, an analog of thymidine readily incorporated into DNA during DNA synthesis. Alive cells were gated and grouped into G0/G1 (quiescent cells or cells preparing for growth), S (cells with active DNA synthesis), and G2/M (cell growth immediately followed by formation of daughter cells) phases together with total DNA content from Hoechst stain (Figure 3E). Treatment with 300 µM BzATP resulted in a significant accumulation of RPMI-8226 in S phase (61.5%, *p* = 0.0014) (Figure 3F). Compared to respective untreated controls, a concomitant reduction was seen in G0/G1 (5.2%, *p* = 0.0086) and G2/M (23.5%, *p* = 0.0015) phases (Figure 3F). Decline in proportion of RPMI-8226 in S phase was detected when performing BrdU labelling after a further 24 h (54.2%) (Figure 3F).

### 3.4. P2X7 Receptor Activation Deregulated Phosphorylation of NF-κB in RPMI-8226

**Figure 4:**
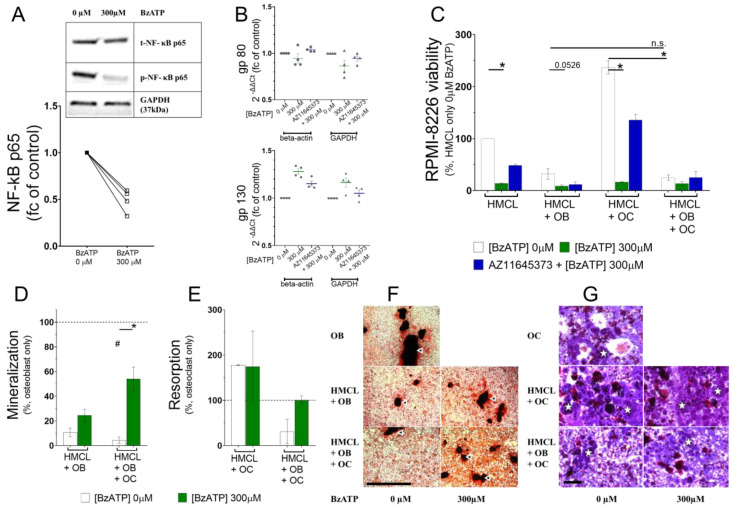
P2X7 receptor activation of transcription regulators and subsequent alterations in RPMI-8226–osteoblast and RPMI-8226–osteoclast interaction. (**A**) Western blot to show the nuclear factor-κappa B (NF-κB) phosphorylation using phosphorylation of NF-κB (p-NF-κB; p65) antibody and quantification of phosphorylated protein normalized to the total protein t-NF-kB (p65) after 90 min treatment with 300 µM BzATP. GAPDH was probed as a protein loading control. (**B**) TaqMan qPCR analysis showing the expression of gp80 and gp130 after 90 min treatment with BzATP (300 µM) alone, or after a 30-min pre-treatment with the antagonist (AZ11645373+ 300 µM), as indicated. Data shows change in mean ± SEM of four independent experiments (*n*) as a fold change of untreated control (0 µM); beta-actin and GAPDH were used as housekeeping genes. (**C**) Effects of RPMI-8266 treatment with 300 µM BzATP for 90 min before culturing with bone cells for 48 h in an in vitro co-culture assay. The effect on viability of RPMI-8266 (HMCL) in the presence of osteoblasts (HMCL+ OB), osteoclasts (HMCL+ OC), or both osteoblasts and osteoclasts (HMCL+ OB + OC) (**D**), and on mineralization and resorption (**E**). Data shows mean ± SEM of independent experiments and * = *p* < 0.05 by Mann–Whitney test comparing the effect of BzATP treatment or by Kruskal–Wallis comparison to compare the effect of bone cells, or # = *p* < 0.05 by Mann–Whitney comparison to baseline bone cell function (dotted line) as indicated (*n* = 3, RPMI-8266 viability; *n* = 4, mineralization; and *n* = 2, resorption). Representative images to show (**F**) Alizarin Red-stained mineralized nodules (white arrows heads) as a measure of osteoblast function (scale bar: 500 µm) and (**G**) resorption (white stars) on dentine discs as a measure of osteoclast function (scale bar: 100 µm; in vitro).

Ultimately, myeloma cell biology is altered by key transcription regulators such as extracellular signal-regulated kinases (ERK1/2), signal transducer and activator of transcription 3 (STAT3), and nuclear factor-κappa B (NF-κB). Treatment with 300 µM BzATP caused a rapid phosphorylation of ERK1/2 (pERK1/2) but phosphorylation of STAT3 (pSTAT3) was reduced in two out of three biological replicates (data not shown). We speculate the inconsistency was due to failing to separate the nuclear and cytosolic components for analysis. It is known that transcriptional regulation, although activated within the nucleus, is often already initiated with the transcription factor bound to cytoplasmic DNA causing a nuclear localization signal prior to targeting the nucleus. Moreover, transcriptional activators associate with other transcriptional co-activator and repressor complexes to ensure long term changes in gene transcription. One such transcriptional activator is p65 subunit of the NF-κB transcription factor complex [34]. We saw a significant reduction in the phosphorylation of p65 after 90 min of 300 µM BzATP in RPMI-8226 (0.49-fold, *p* = 0.0286) (Figure 4A).

Phosphorylation of p65 subunit has received the most attention in studies of NF-κB phosphorylation thus far. p65 heightens inflammatory cytokine production in myeloma bone disease, such as the release of interleukin-6 (IL-6) by associating with members of the IL-6 signaling complex [35]. IL-6 signal transduction relies on co-expression of two transmembrane molecules (gp80 and gp130) to constitute a functional IL-6 receptor (IL-6R). The smaller subunit (gp80) must associate with a larger structure of gp130 for high affinity binding and IL-6 signaling. On the contrary, gp130 alone acts as a signal transducer that is responsible for a variety of biological activities [36]. gp130 signaling is an important driver of MM pathogenesis [37,38]. RPMI-8226 express both gp80 and gp130 while IL-6 is absent [39,40]. We tested if the components of IL-6 signal transduction are regulated by the P2X7 receptor. RPMI-8226 were treated with 300 µM BzATP and the supernatant was collected at time intervals. IL-6 secretion was undetected by ELISA even at 48 h, which corresponded to lower mRNA copy numbers of IL-6 transcript in RPMI-8226 cells (> 35 Ct values, data not shown). Both gp80 (IL-6R) and gp130 transcripts were regulated after P2X7 receptor activation. Downregulation of gp80 (0.95-fold and 0.86-fold normalized to housekeeping gene beta-actin and GAPDH, respectively) and upregulation of gp130 (1.28-fold and 1.19-fold normalized to housekeeping gene beta-actin and GAPDH, respectively) after P2X7 receptor activation RPMI-8226 was observed (Figure 4B).

### 3.5. P2X7 Receptor Restricted RPMI-8226 Growth even in the Presence of the Bone Cells and Altered RPMI-8226–Osteoblast and RPMI-8226–Osteoclast Interactions

Targeting the bone–myeloma interdependence has been explored with interest as a therapy for MM patients. We explored whether P2X7 receptor has consequences for the myeloma–bone interaction using a co-culture assay to assess four conditions: RPMI-8226 (1) treated with 300 µM BzATP for 90 min, with or without a 30 min antagonist pretreatment (HMCL), (2) treated and then co-cultured with mature primary osteoblasts (HMCL+OB), (3) treated and then co-cultured with resorbing osteoclasts (HMCL+OC), and (4) treated and then co-cultured with both bone cells (HMCL + OB + OC). All analyses were performed after 2 days.

First, a regulation of RPMI-8226 viability by bone cells was seen in our 3D co-culture model. Osteoblasts restricted RPMI-8226 proliferation (31.93% HMCL + OB versus HMCL, white) (Figure 4C) while osteoclasts favored it (237% HMCL + OC versus HMCL, white). Osteoblasts and osteoclasts (HMCL + OB + OC) reduced RPMI-8226 viability to 24.95% compared to the RPMI-8226 monoculture (Table 2). We next determined whether direct effects of P2X7 receptor activation observed previously (Figure 3A) were maintained. Significant reduction in RPMI-8226 viability with 300 µM BzATP (13.78%, *p* = 0.0070, green) and partial reversal with antagonist pretreatment (48.47%, blue) confirmed the P2X7 receptor-mediated effect on RPMI-8226 (Figure 4C, Table 2). Most interestingly, primary bone cells altered the behavior of activated RPMI-8226 when compared to the non-activated controls. Osteoblasts (HMCL + OB) synergized the effect of P2X7 receptor as viability of RPMI-8226 was further inhibited (8.81% with 300 µM BzATP, *p* = 0.0526 versus 31.93% with 0 µM BzATP). However, this inhibition was presumably due to a dominant osteoblastic effect and was not mediated by P2X7 receptor, as any reversal with antagonist pretreatment of RPMI-8226 was lacking (11.71% with 1 µM AZ11645373). On the other hand, osteoclasts no longer maintained a pro-myeloma effect on activated RPMI-8226 (16.59% with 300 µM BzATP, *p* = 0.0073 versus 237% with 0 µM BzATP) but could be prevented with P2X7 receptor antagonist (135.8% with 1 µM AZ11645373) (Figure 4C). Presence of both osteoblasts and osteoclasts (HMCL + OB + OC) reduced RPMI-8226 viability (13.33% with 300 µM BzATP versus 25% with 0 µM BzATP) and was prevented by P2X7 receptor antagonist (25% with 1 µM AZ11645373), but these findings did not reach statistical significance (Table 2). Together, these data show that osteoblasts exerted a dominant effect on RPMI-8226 inhibition, which, in spite of an osteoclastic growth promotion of myeloma cells, was synergistic to the P2X7 receptor-induced growth arrest.

Next, we examined if P2X7 receptor alters the interaction of RPMI-8226 with bone cells as interrupting the myeloma-bone interdependence is imperative to disrupt metabolic bone disease. The extent of mineral deposition provides valuable insight about the process of bone formation and acts as a measure of osteoblast function in vitro. Osteoblastic mineralization was reduced in the presence of RPMI-8226 (10.7% of baseline, dotted line, *p* = 0.0579), which remained suppressed with their P2X7 receptor activation (24.57%, green bar HMCL + OB) (Figure 4D). Triple culture with osteoclasts (HMCL + OB + OC) did not affect the RPMI-8226-mediated inhibition of osteoblastic mineralization (4.44% of baseline, dotted line, *p* = 0.0038). However, osteoblast function was uplifted with P2X7 receptor activation of RPMI-8226 (53.94% versus 0 µM BzATP, *p* = 0.0286) (Figure 4D). These results show that P2X7 receptor induces a growth arrest in RPMI-8226 and the presence of osteoclasts is effective in restoring the myeloma-induced inhibition of osteoblastic function.

To assess osteoclast function, we evaluation percentage of resorption of a bone substrate. Increased osteoclastic resorption in the presence of RPMI-8226 (177.9% of baseline, dotted line) remained unchanged with their P2X7 receptor activation (174.3%, green bar Figure 4D) (HMCL + OC). Presence of osteoblasts (HMCL + OB + OC) diminished the RPMI-8226-mediated pro-osteoclastic effect (31.09% resorption of baseline, dotted line). However, osteoclast function was restored in the presence of RPMI-8226 previously treated with 300 µM BzATP (100.8% versus 0 µM, Figure 4E). In all, while osteoclast function was heightened by RPMI-8226, the excessive resorption was prevented by their P2X7 receptor when osteoblasts were also present.

## 4. Discussion

P2X7 receptor has an ambiguous role in cancer progression with both pro- and anti-cancerous effects, depending on the cancer or tumor cell type. Its role in myeloma biology and relevance in the consequent bone disease is not defined. We used RPMI-8226 to show that human P2X7 receptor induces S phase cell cycle arrest and alters transcriptional regulation to reduce the myeloma growth. Further, our co-culture model demonstrated in vitro that P2X7 receptor regulates the crosstalk of RPMI-8226 with osteoblast and that a synergistic bone anabolic therapy can be explored. We also speculate that the reversal of hyperactive osteoclast function in vitro represents a normalized bone turnover, mitigating the pathology of the bone disease.

Our exploratory findings provide a rationale for advancing the research in P2X7 receptor-mediated MM progression. A functional P2X7 receptor is seen by conventional methods such as (a) membrane permeability and (b) Ca^2+^ influx following agonist stimulus on HMCLs. These HMCLs represent diversity of tissue origin and patient disease stage, which may explain the differences in agonist potency. These differences in pharmacology are highly relevant for translation into the clinic as the diversity of clones in metastatic diseases is known to affect responses to therapies. Pre-clinical models reflecting patient-to-patient variability are highly warranted to address the superiority of P2X7 receptor as an independent drug target, or in synergy with drug combinations, in MM patients. Additional complexity to the regulatory functions of P2X7 receptor is provided by the highly polymorphic human P2X7 receptor gene (*P2RX7).* Several single nucleotide polymorphisms (SNPs) increase or decrease association to disease susceptibility [41]. An association to the risk of myeloma is described with contrasting results [42,43]. Furthermore, the two isoforms P2X7A and P2X7B impart different functional properties [28], and co-expression of both is required for the P2X7 receptor membranal pores. It is speculated that a homo/heteromeric P2X7A/B receptor may be advantageous to cancer cells during increased eATP [44] and a ligand-induced acquisition of varying stoichiometry of subunits may determine cell survival/death [30]. Moreover, RPMI-8226 are capable of active P2X7 receptor modulation, as expression increases to several-fold when exposed to high eATP [45]. We saw a strong immunoreactivity to both P2X7 receptor antibodies, implying a functional protein composed of both P2X7A and B subunits (Figure 2A). Thus, depending on the stimulus, a ligand-induced P2X7 receptor modulation may support their growth or trigger death. We observed a RPMI-8226 cycle arrest as one of the events (Figure 3F). Thus far, P2X7 receptor-mediated shedding of cell surface molecules CD23 and CXCL16 with cytokine-like activities, as well as a loss of plasma membrane integrity, are indicated in RPMI-8226 [20,21]. While an increase in circulating levels of CD23 and CXCL16 (both being cell membrane bound signaling proteins) may have consequences in the pathology of MM disease, induction of cell death/necrosis seems counter-intuitive for progression. RPMI-8226 show significant decline in cell viability without a reduction in cell numbers (Figure 3A,B). A consistent induction of apoptosis and necrosis was not achieved in our study, and even caspase-3 cleavage was reduced. P2X7 receptor activation could have triggered a “danger signal” where cell cycle arrest is used as a protective mechanism. Downregulation of p65 NF-κB signaling also indicated a protective mechanism by suppressing proliferation (Figure 4C). We acknowledge that these effects are transient, as activation of NF-κB, ERK1/2, and STAT3 signaling are critical growth and survival pathways during drug-induced apoptosis. Aligning with this, S phase arrest was already lifted at the 72 h timepoint (Figure 3F). Nevertheless, P2X7 receptor activation may induce P2X7 subunit modulation and thus an expansion of RPMI-8226 clones with a new phenotype. These resultant clones may be resistant and desensitized to a similar signal, thus acquiring a survival benefit. One of the mechanisms by which myeloma cells develop multi-drug resistance is associated with poor prognosis in patients.

Homeostatic bone turnover requires a precise activity between the bone cells and MM patients present with a severe bone destruction. Myeloma-derived factors cause hyperactivation of osteoclasts and suppression of osteoblast differentiation orchestrated by paracrine and endocrine factors such as cytokines and various signaling molecules [46,47]. Our co-culture model helps understand the direct interaction between these three cells, shielded from any external regulation. We not only found an osteoblastic suppression and osteoclastic hyperactivation of RPMI-8226 (Figure 4C), but also a dominant osteoblastic suppression, regardless of a pro-myeloma osteoclastic effect. The former was exacerbated via P2X7 receptor. We speculate that a temporary arrest of RPMI-8226 growth assists the osteoblast inhibition, but more studies are needed to confirm a long-term effect. Moreover, P2X7 receptor regulates the lifespan and activity of osteoclasts and inhibits osteoblast function [48,49]. This potential for P2X7 receptor may support the case for an anabolic/dual bone therapy in MM patients and will influence overall bone turnover in patients with an active bone disease.

In conclusion, we show the variations in P2X7 receptor structure in myeloma cells as well as striking pharmacological differences, particularly in terms of the agonist profile. Although the signaling and growth factors require further investigation, we surmise that P2X7 receptor directly influences the myeloma–osteoblast and myeloma–osteoclast cross talk and therefore acknowledge the potential of P2X7 receptor as a target in MBD. Our study lacks an in vivo model supporting this potential, and pre-clinical investigations are needed to have a relevant transitional effect on patients. A heterogeneity in P2X7 receptor expression, potential of subunit co-assembly, and eATP-driven modulation of P2X7 receptor isoforms may all be important determinants in clonal progression. To have a therapeutic effect, an ideal modulator would be selective between the P2X7 receptor variants such as the blocking antibody targeted against a non-functional P2X7 receptor epitope currently being explored in clinical trials [50]. The time for intervention will also be key in the feasibility of the P2X7 receptor as a therapy for MM patients. Equally imperative will be to understand the effect on healthy cell subsets, but ATP administration appears to be a promising strategy in leukemia treatment [51]. Lastly, as alleles imparting a loss of function to the gene for human P2X7 receptor are associated with an increased risk of MM, our findings support that a complete activation of P2X7 receptor is more relevant in the pathology of MM and the eventual treatment of myeloma-induced bone disease. Therefore, a potential therapy will involve a personalized, timely, and limited intervention with a P2X7 receptor modulator, potentially in combination with an existing treatment.

## Figures and Tables

**Table 1:** Summary of P2X7 receptor pore formation and channel function induced by BzATP in all HMCLs.

	RPMI-8226	U226	NCI-H929	OPM2	JJN3	KMS12
**AUC of maximum, % mean ± SEM (n)**						
0 µM	32.03 ± 3.25 (9)	14.21 ± 0.74 (9)	16.10 ± 1.32 (9)	33.21 ± 6.40 (7)	47.96 ± 8.81 (7)	17.06 ± 1.52 (7)
500 µM	64.92 ± 3.16 (9)	20.11 ± 1.48 (9)	17.82 ± 1.41 (9)	44.95 ± 6.94 (7)	49.59 ± 7.66 (7)	23.82 ± 2.44 (7)
*p*-Value	**0.0067**	**0.0203**	0.5092	0.1622	0.8143	0.1081
10 µM KN62 + 500 µM BzATP	41.98 ± 3.28 (2)	16.35 ± 0.89 (2)	18.64 ± 1.58 (2)	43.20 ± 7.16 (6)	40.70 ± 6.47 (6)	21.30 ± 2.48 (6)
*p*-Value	0.3615	0.5025	0.7681	0.5836	0.5300	0.6135
10 µM A740003 + 500 µM BzATP	31.58 ± 1.62 (2)	16.50 ± 0.68 (2)	19.57 ± 2.37 (2)	45.42 ± 8.11 (6)	44.95 ± 8.22 (6)	21.60 ± 2.02 (6)
*p*-Value	0.1688	0.5025	0.6214	0.6810	0.7351	0.6227
10 µM AZ11645373 + 500 µM BzATP	25.94 ± 1.29 (2)	12.51 ± 0.21 (2)	13.35 ± 0.74 (2)	41.73 ± 6.38 (6)	37.61 ± 7.24 (6)	25.65 ± 3.08 (6)
*p*-Value	**0.0353**	**0.0459**	0.3020	0.5107	0.3190	0.6495
F_max_/F_0_, mean ± SEM (n)						
0 µM BzATP	0.678 ± 0.019 (6)	0.516 ± 0.141 (7)	0.598 ± 0.033 (7)	0.737 ± 0.077 (5)	0.803 ± 0.120 (7)	0.840 ± 0.072 (7)
100 µM BzATP	1.668 ± 0.146 (6)	0.916 ± 0.027 (7)	1.056 ± 0.062 (7)	1.845 ± 0.108 (5)	1.136 ± 0.089 (7)	1.647 ± 0.069 (7)
*p*-Value	**0.0008**	**0.0041**	**0.0001**	**0.0009**	**0.0413**	**0.0002**
1 µM KN62 + 100 µM BzATP	0.853 ± 0.258 (2)	0.853 ± 0.041 (3)	0.905 ± 0.124 (3)	1.668 ± 0.117 (5)	0.994 ± 0.073 (5)	1.397 ± 0.087 (5)
*p*-Value	**0.0389**	0.4791	0.1355	0.4143	0.2287	0.1818
1 µM A740003 + 100 µM BzATP	0.753 ± 0.102 (2)	0.988 ± 0.035 (3)	1.017 ± 0.143 (3)	1.758 ± 0.089 (5)	1.054 ± 0.092 (5)	1.539 ± 0.189 (5)
*p*-Value	**0.0389**	0.3389	0.4908	0.7310	0.5473	0.6221
1 µM AZ11645373 + 100 µM BzATP	0.890 ± 0.010 (2)	0.940 ± 0.016 (3)	0.926 ± 0.166 (3)	1.544 ± 0.120 (5)	1.103 ± 0.082 (5)	1.580 ± 0.112 (5)
*p*-Value	0.2513	0.9847	0.1079	0.1829	0.8098	0.8009
	Bold represents significant *p*-Value					

**Table 2:** Effect of P2X7 receptor activation of RPMI-8266 interaction with osteoblasts and osteoclasts in 3-cell culture set up in vitro.

	HMCL	HMCL + OB	*p*-Value	HMCL + OC	*p*-Value	HMCL + OB + OC	*p*-Value
Viability, % mean ± SEM (n = 3)							
0 µM	100 ± 0.01	31.93 ± 10.08	0.1403	237.0 ± 12.75	0.3073	24.95 ± 5.31	0.1123
300 µM	13.78 ± 0.79	8.81 ± 2.12		16.59 ± 0.74		13.33 ± 3.63	
*p*-value	**0.0070**	0.0526		**0.0073**		0.1797	
10 µM AZ11645373 + 300 µM BzATP	48.47 ± 2.30	11.71 ± 4.83		135.8 ± 10.46		25.20 ± 11.54	
*p*-value	0.1779	0.4561		0.1797		0.3711	
Mineralization, % of OB mean ± SEM (n = 4)							
0 µM	-	10.65 ± 3.54	0.0579	-		4.44 ± 2.54	**0.0038**
300 µM		24.57 ± 4.91				53.94 ± 9.84	
*p*-value		0.1143				**0.0286**	
Resorption, % of OC mean ± SEM (n = 2)							
0 µM	-	-		177.9 ± 1.36	0.2781	31.09 ± 27.21	0.2781
300 µM				174.3 ± 78.82		100.8 ± 8.72	
*p*-value				>0.999		0.333	
	Bold represents significant *p*-Value						

## Supplementary Materials

The following are available online at https://www.mdpi.com/2073-4409/9/11/2341/s1, Figure S1: setting for triple cell culture system.

## References

[1] Marino S., Roodman G.D. (2018). Multiple Myeloma and Bone: The Fatal Interaction. Cold Spring Harb. Perspect. Med..

[2] Silbermann R., Roodman G.D. (2016). Current Controversies in the Management of Myeloma Bone Disease. J. Cell Physiol..

[3] Kawano Y., Moschetta M., Manier S., Glavey S., Gorgun G.T., Roccaro A.M., Anderson K.C., Ghobrial I.M. (2015). Targeting the bone marrow microenvironment in multiple myeloma. Immunol. Rev..

[4] Burnstock G. (2018). Purine and purinergic receptors. Brain Neurosci. Adv..

[5] Burnstock G., Verkhratsky A. (2010). Long-term (trophic) purinergic signalling: Purinoceptors control cell proliferation, differentiation and death. Cell Death Dis..

[6] De Andrade Mello P., Coutinho-Silva R., Savio L.E.B. (2017). Multifaceted effects of extracellular adenosine triphosphate and adenosine in the Tumor-Host interaction and therapeutic perspectives. Front. Immunol..

[7] Di Virgilio F. (2016). P2RX7: A receptor with a split personality in inflammation and cancer. Mol. Cell. Oncol..

[8] Helenius M., Jalkanen S., Yegutkin G. (2012). Enzyme-coupled assays for simultaneous detection of nanomolar ATP, ADP, AMP, adenosine, inosine and pyrophosphate concentrations in extracellular fluids. Biochim. Biophys. Acta.

[9] Joseph S.M., Buchakjian M.R., Dubyak G.R. (2003). Colocalization of ATP release sites and ecto-ATPase activity at the extracellular surface of human astrocytes. J. Biol. Chem..

[10] Pellegatti P., Raffaghello L., Bianchi G., Piccardi F., Pistoia V., Di Virgilio F. (2008). Increased level of extracellular ATP at tumor sites: In vivo imaging with plasma membrane luciferase. PLoS ONE.

[11] Di Virgilio F., Pinton P., Falzoni S. (2016). Assessing Extracellular ATP as Danger Signal In Vivo: The pmeLuc System. Methods Mol. Biol..

[12] Burnstock G. (2020). Introduction to Purinergic Signaling. Methods Mol. Biol..

[13] Di Virgilio F., Sarti A.C., Falzoni S., De Marchi E., Adinolfi E. (2018). Extracellular ATP and P2 purinergic signalling in the tumour microenvironment. Nat. Rev. Cancer.

[14] Burnstock G., Knight G.E. (2018). The potential of P2X7 receptors as a therapeutic target, including inflammation and tumour progression. Purinergic Signal..

[15] Lara R., Adinolfi E., Harwood C.A., Philpott M., Barden J.A., Di Virgilio F., McNulty S. (2020). P2X7 in Cancer: From Molecular Mechanisms to Therapeutics. Front. Pharm..

[16] Adinolfi E., Capece M., Franceschini A., Falzoni S., Giuliani A.L., Rotondo A., Sarti A.C., Bonora M., Syberg S., Corigliano D. (2015). Accelerated tumor progression in mice lacking the ATP receptor P2X7. Cancer Res..

[17] Adinolfi E., Raffaghello L., Giuliani A.L., Cavazzini L., Capece M., Chiozzi P., Bianchi G., Kroemer G., Pistoia V., Di Virgilio F. (2012). Expression of P2X7 receptor increases in vivo tumor growth. Cancer Res..

[18] Di Virgilio F., Adinolfi E. (2017). Extracellular purines, purinergic receptors and tumor growth. Oncogene.

[19] Gorodeski G.I. (2009). P2X7 -mediated chemoprevention of epithelial cancers. Expert Opin. Targets.

[20] Farrell A.W., Gadeock S., Pupovac A., Wang B., Jalilian I., Ranson M., Sluyter R. (2010). P2X7 receptor activation induces cell death and CD23 shedding in human RPMI 8226 multiple myeloma cells. Biochim. Biophys. Acta Gen. Subj..

[21] Pupovac A., Foster C.M., Sluyter R. (2013). Human P2X7 receptor activation induces the rapid shedding of CXCL16. Biochem. Biophys. Res. Commun..

[22] Pupovac A., Geraghty N.J., Watson D., Sluyter R. (2015). Activation of the P2X7 receptor induces the rapid shedding of CD23 from human and murine B cells. Immunol. Cell Biol..

[23] Falk S., Schwab S.D., Frosig-Jorgensen M., Clausen R.P., Dickenson A.H., Heegaard A.M. (2015). P2X7 receptor-mediated analgesia in cancer-induced bone pain. Neuroscience.

[24] Falk S., Appel C.K., Bennedbaek H.B., Al-Dihaissy T., Unger A., Dinkel K., Heegaard A.M. (2019). Chronic high dose P2X7 receptor inhibition exacerbates cancer-induced bone pain. Eur. J. Pharm..

[25] Burnstock G. (2016). Purinergic Mechanisms and Pain. Adv. Pharm..

[26] Agrawal A., Henriksen Z., Syberg S., Petersen S., Aslan D., Solgaard M., Nissen N., Larsen T.K., Schwarz P., Steinberg T.H. (2017). P2X7Rs are involved in cell death, growth and cellular signaling in primary human osteoblasts. Bone.

[27] Agrawal A., Gallagher J.A., Gartland A. (2012). Human osteoclast culture and phenotypic characterization. Methods Mol. Biol..

[28] Giuliani A.L., Colognesi D., Ricco T., Roncato C., Capece M., Amoroso F., Wang Q.G., De Marchi E., Gartland A., Di Virgilio F. (2014). Trophic activity of human P2X7 receptor isoforms A and B in osteosarcoma. PLoS ONE.

[29] Buell G., Chessell I.P., Michel A.D., Collo G., Salazzo M., Herren S., Gretener D., Grahames C., Kaur R., Kosco-Vilbois M.H. (1998). Blockade of human P2X7 receptor function with a monoclonal antibody. Blood.

[30] Adinolfi E., Cirillo M., Woltersdorf R., Falzoni S., Chiozzi P., Pellegatti P., Callegari M.G., Sandona D., Markwardt F., Schmalzing G. (2010). Trophic activity of a naturally occurring truncated isoform of the P2X7 receptor. FASEB J..

[31] Cheewatrakoolpong B., Gilchrest H., Anthes J.C., Greenfeder S. (2005). Identification and characterization of splice variants of the human P2X7 ATP channel. Biochem. Biophys. Res. Commun..

[32] Feng Y.H., Li X., Wang L., Zhou L., Gorodeski G.I. (2006). A truncated P2X7 receptor variant (P2X7-j) endogenously expressed in cervical cancer cells antagonizes the full-length P2X7 receptor through hetero-oligomerization. J. Biol. Chem..

[33] Liang X., Samways D.S., Wolf K., Bowles E.A., Richards J.P., Bruno J., Dutertre S., DiPaolo R.J., Egan T.M. (2015). Quantifying Ca2+ current and permeability in ATP-gated P2X7 receptors. J. Biol. Chem..

[34] Christian F., Smith E.L., Carmody R.J. (2016). The Regulation of NF-kappaB Subunits by Phosphorylation. Cells.

[35] Giuliani N., Colla S., Morandi F., Rizzoli V. (2004). The RANK/RANK ligand system is involved in interleukin-6 and interleukin-11 up-regulation by human myeloma cells in the bone marrow microenvironment. Haematologica.

[36] Mihara M., Hashizume M., Yoshida H., Suzuki M., Shiina M. (2012). IL-6/IL-6 receptor system and its role in physiological and pathological conditions. Clin. Sci. (Lond).

[37] Dechow T., Steidle S., Gotze K.S., Rudelius M., Behnke K., Pechloff K., Kratzat S., Bullinger L., Fend F., Soberon V. (2014). GP130 activation induces myeloma and collaborates with MYC. J. Clin. Investig..

[38] Burger R., Gunther A., Klausz K., Staudinger M., Peipp M., Penas E.M., Rose-John S., Wijdenes J., Gramatzki M. (2017). Due to interleukin-6 type cytokine redundancy only glycoprotein 130 receptor blockade efficiently inhibits myeloma growth. Haematologica.

[39] Gougelet A., Mansuy A., Blay J.Y., Alberti L., Vermot-Desroches C. (2009). Lymphoma and myeloma cell resistance to cytotoxic agents and ionizing radiations is not affected by exposure to anti-IL-6 antibody. PLoS ONE.

[40] Schwabe M., Brini A.T., Bosco M.C., Rubboli F., Egawa M., Zhao J., Princler G.L., Kung H.F. (1994). Disruption by interferon-alpha of an autocrine interleukin-6 growth loop in IL-6-dependent U266 myeloma cells by homologous and heterologous down-regulation of the IL-6 receptor alpha- and beta-chains. J. Clin. Investig..

[41] Sluyter R., Stokes L. (2011). Significance of P2X7 receptor variants to human health and disease. Recent Pat. Dna Gene Seq..

[42] Paneesha S., Starczynski J., Pepper C., Delgado J., Hooper L., Fegan C., Pratt G. (2006). The P2X7 receptor gene polymorphism 1513 A-->C has no effect on clinical prognostic markers and survival in multiple myeloma. Leuk. Lymphoma.

[43] Vangsted A.J., Klausen T.W., Gimsing P., Abildgaard N., Andersen N.F., Gang A.O., Holmstrom M., Gregersen H., Vogel U., Schwarz P. (2014). Genetic variants in the P2RX7 gene are associated with risk of multiple myeloma. Eur. J. Haematol..

[44] Adinolfi E., Callegari M.G., Ferrari D., Bolognesi C., Minelli M., Wieckowski M.R., Pinton P., Rizzuto R., Di Virgilio F. (2005). Basal activation of the P2X7 ATP receptor elevates mitochondrial calcium and potential, increases cellular ATP levels, and promotes serum-independent growth. Mol. Biol. Cell.

[45] Gilbert S.M., Oliphant C.J., Hassan S., Peille A.L., Bronsert P., Falzoni S., Di Virgilio F., McNulty S., Lara R. (2019). ATP in the tumour microenvironment drives expression of nf P2X7, a key mediator of cancer cell survival. Oncogene.

[46] Shupp A.B., Kolb A.D., Mukhopadhyay D., Bussard K.M. (2018). Cancer Metastases to Bone: Concepts, Mechanisms, and Interactions with Bone Osteoblasts. Cancers.

[47] Walker R.E., Lawson M.A., Buckle C.H., Snowden J.A., Chantry A.D. (2014). Myeloma bone disease: Pathogenesis, current treatments and future targets. Br. Med. Bull..

[48] Agrawal A., Gartland A. (2015). P2X7 receptors: Role in bone cell formation and function. J. Mol. Endocrinol..

[49] Wang N., Agrawal A., Jorgensen N.R., Gartland A. (2018). P2X7 receptor regulates osteoclast function and bone loss in a mouse model of osteoporosis. Sci. Rep..

[50] Gilbert S.M., Gidley Baird A., Glazer S., Barden J.A., Glazer A., Teh L.C., King J. (2017). A phase I clinical trial demonstrates that nf P2X7 -targeted antibodies provide a novel, safe and tolerable topical therapy for basal cell carcinoma. Br. J. Derm..

[51] Salvestrini V., Orecchioni S., Talarico G., Reggiani F., Mazzetti C., Bertolini F., Orioli E., Adinolfi E., Di Virgilio F., Pezzi A. (2017). Extracellular ATP induces apoptosis through P2X7R activation in acute myeloid leukemia cells but not in normal hematopoietic stem cells. Oncotarget.

