# Supplementary material for: Human P2X7 Receptor Causes Cycle Arrest in RPMI-8226 Myeloma Cells to Alter the Interaction with Osteoblasts and Osteoclasts
Source: Cells. 2020 Oct 22;9(11):2341. doi: 10.3390/cells9112341 (PMC7690412; doi:10.3390/cells9112341)
Supplement: Supplementary file 1 [file cells-09-02341-s001.pdf]

Supplementary Information

Human P2X7 receptor causes cycle arrest in RPMI-8226 myeloma cells to alter the interaction with osteoblasts and osteoclasts

Ankita Agrawal[1], Lars S. Kruse, Annette J. Vangsted[2], Alison Gartland[3], and Niklas R. Jørgensen[1,4]

[1]Department of Clinical Biochemistry, Copenhagen University Hospital Rigshospitalet, Denmark.

[2]Department of Hematology, Copenhagen University Hospital, Rigshospitalet, Denmark.

[3]The Mellanby Centre for Musculoskeletal Research, Department of Oncology and Metabolism, The University of Sheffield, Sheffield UK.

[4]Department of Clinical Medicine, Faculty of Health and Medical Sciences, University of Copenhagen, Copenhagen, Denmark.

Materials and Methods

1. *Cell culture and reagents*

RPMI-8226, U266, NCI-H929, and KMS12 were maintained at 37°C and 5% CO_2 in RPMI-1620 medium (Fisher Scientific, Roskilde, Denmark) supplemented with 10% heat-inactivated fetal bovine serum (h.i. FBS; Sigma-Aldrich, Soeborg, Denmark), 1mM sodium pyruvate (Fisher Scientific), 1x MEM Non-Essential Amino Acids Solution (Fisher Scientific), 100 U/mL penicillin, and 100 µg/mL streptomycin (Fisher Scientific). For OPM2, RPMI-1620 medium was supplemented with 10% h.i. FBS, 1mM sodium pyruvate, 100 U/mL penicillin, and 100 µg/mL streptomycin and JJN3 medium was 40% Dulbecco's MEM + 40% Iscove's MDM + 20% h.i. FBS. All cells were maintained at densities between 0.4 and 1 × 10^6 cells/mL by subculturing, as recommended by ATCC and/or DSMG.

Primary osteoblasts were obtained from human trabecular bone explants, as previously described [26]. Primary osteoclasts were differentiated from peripheral blood monocytes at 37°C and 7% CO_2 in αMEM medium (Fisher Scientific) by CD14+ enrichment using RosetteSep human monocyte enrichment cocktail (STEMCELL Technologies, Cambridge, United Kingdom) according to the manufacturer's instructions. Primary samples were collected after approval from local ethics committees (H-1-2013-003 and H-1-2013-002).

The synthetic competitive P2X7R agonist, benzoylated ATP (BzATP), and P2X7R blockers (KN62: first generation, non-competitive antagonist; A740003: non-competitive/allosteric binding site; and AZ11645373: newest generation, non-competitive antagonist), probenecid, and DMSO were purchased from Sigma-Aldrich. YO-PRO-1, Fluo-4-AM, Pluronic-F 127,

Quant-iT PicoGreen dsDNA Assay Kit, Dead Cell Apoptosis Kit with annexin V and propidium iodide (PI), and goat anti-mouse Alexa Fluor 488 were from Fisher Scientific. Bromodeoxyuridine (BrdU), Alexa Fluor 488 mouse anti-BrdU, and DAPI were from BD Biosciences (Becton Dickinson A/S, Denmark). Bortezomib (10 mM/ mL) was provided in DMSO as solvent (Selleck chemicals, Rungsted, Denmark) and stored in -20°C until use. The anti-P2X7R antibody P2X7R-ec was a kind gift from Dr. Iain Chessell, and P2X7R-Cter (APR-004 and peptide antigen) was from Alomone Labs (Jerusalem, Israel). Anti-GAPDH was from Cell Signaling Technology (#5174, BioNordika Denmark A/S), while secondary antibodies were anti-rabbit IgG (NA934, GE Healthcare) and anti-mouse IgG (Fisher Scientific).

2. *Genotyping*

HMCL were lysed using 25mM NaOH/0.2 mM EDTA and neutralized with 40 mM Tris-HCl buffer. Genomic DNA was precipitated with 70% ethanol and dissolved in water before storing at -20°C. DNA concentration was determined using PicoGreen assay and integrity was checked by electrophoresis on a 0.8% agarose gel. After adjusting the concentration to 5 ng/ μL, we analyzed genotyping for the non-synonymous single nucleotide polymorphisms (SNPs) with functional consequences at LGC Genomics Ltd (Hoddesdon, United Kingdom), as previously published [28].

3. *Semiquantitative PCR*

Total RNA was obtained with an RNeasy Mini Kit (Qiagen Nordic, Sweden) according to the manufacturer's instructions, and concentration was determined using a Nanodrop 2000 Spectrophotometer (Fisher Scientific). After assessing the integrity of each sample with the Eukaryotic 2100 bioanalyzer (Agilent Technologies Denmark A/S), we synthesized cDNA from 2 μg total RNA using the High Capacity cDNA Reverse Transcription Kit (Applied Biosystems, Fisher Scientific) according to the manufacturer's instructions. The semiquantitative RT-PCR used previously published primers [27,30]: forward 5′ AGATCGTGGAGAATGGAGTG 3′, reverse 5′ TTCTCGTGGTGTAGTTGTGG 3′ for pan P2X7; forward 5′ AGATGCTGGAGAATGGAGTG 3′, reverse 5′ TTCTCGTGGTGTAGTTGTGG 3′ for P2X7A; forward 5′ CCCATCGAGGCAGTGGA 3′, reverse 5′ TAAAGCATGGAAAAGAGAATCTC 3′ for P2X7B/J; and forward 5′ CCTCTGACTTCAACAGCGAC, 3′ reverse 5′ CATGACAAGGTGCGGCTCCC 3′ for G3PDH. The PCR conditions were heat activation of HotStarTaq DNA Polymerase (95°C for 15 minutes) followed by 40 cycles each of denaturation (95°C for 1 min), annealing (50°C for 1 minute), and extension (68°C for 2 minutes). Final extension was achieved at 72°C for 10 minutes. All products were electrophoresed on 1% agarose gel and visualized under UV light.

4. *Pore formation assay*

Uptake of YO-PRO-1 (molecular weight: 629 Da) was used to measure pore formation. HMCL were seeded in poly-D-lysine-coated clear-bottom black plates at a density of 10^5 cells per well, 48 hours prior to use. On the day of the assay, cells were rinsed in HBSS without Ca^{2+}

and Mg^{2+} containing 20 mM HEPES (pH 7.4; assay buffer). After the cells were equilibrated for 10 minutes at 37°C/5% CO_2 in YO-PRO-1 (2 μM) containing assay buffer, we recorded fluorescence (491/ 509) by NOVOstar (BMG Labtech, Ramcon A/S Denmark). For antagonist activity, all compounds (or DMSO) were added at the same time as YO-PRO-1 before inducing P2X7R-dependent pore formation using 500 μM BzATP. At the end of each time course, cell lysis buffer was added to induce maximal YO-PRO-1 uptake. After adjusting for auto-fluorescence, obtained by injecting assay buffer in empty wells, we used area under the curve (AUC) for quantitative analysis.

5. *Measurement of intracellular calcium concentration*

Change in intracellular calcium concentration $[Ca^{2+}]_i$ was measured by fluorescence of the Ca^{2+}-intercalating dye Fluo-4 AM. HMCLs were seeded in poly-D-lysine-coated clear-bottom black plates at a density of 10^5 cells per well, 48 hours prior to use. On the day of the assay, cells were incubated with Fluo-4 AM (4 μM containing 50 % v/v Pluronic F-127) in HBSS containing 20 mM HEPES, 2 mM $CaCl_2$, 0.5 mM $MgCl_2$, and 2.5 mM Probenecid (pH 7.4; assay buffer) in the dark for 1 hour at 37°C/ 5% CO_2. Cells were rinsed in warm assay buffer and equilibrated for 10 minutes before fluorescence measurement for 10 seconds (baseline) (Ex/Em of Ca^{2+}-bound Fluo-4 485/520 nm). For measurement of antagonist activity, compounds (or DMSO) were introduced with Fluo-4 AM before activation with BzATP (100 μM). A calcium ionophore A23187 (50 μM) was used to saturate Fluo-4 with free Ca^{2+} at the end of each measurement. After adjusting for auto-fluorescence, obtained by injecting assay buffer in empty wells, we used peak fluorescence as a fold change from baseline for quantitative analysis.

6. *Detection of P2X7 receptor protein*

Total protein was obtained by lysing RPMI-8226 in ice-cold buffer (100 mM Tris-HCl (pH 7.5), 10 mM EDTA, 10% Triton X, 100 mM NaF, and phosphatase and protease inhibitors). After sonication and removal of sub-cellular debris by centrifugation, we determined protein content of the extracts by DC protein assay kit (Bio-Rad Laboratories, Copenhagen, Denmark). Equal amounts of total protein extracts (25 μg) were subjected to denaturing electrophoresis on Mini-PROTEAN Tris-Glycine eXtended gels. Proteins were transferred to a 0.2 μm polyvinylidene fluoride (PVDF) membrane using the Trans-Blot Turbo System before incubating with either anti-P2X7R-Cter antibody (0.6 μg/ml, control-included peptide antigen at 1 μg peptide per 1 μg antibody) or anti-P2X7R-ec (1.15 μg/mL) overnight at 4°C. Anti-GAPDH (#D16H11) was used as a control for sample loading. Anti-rabbit IgG- and anti-mouse IgG-conjugated to HRP were used for detection of anti-P2X7R-Cter and anti-P2X7R-ec antibodies for 1 hour at room temperature (RT), respectively. Immunodetection was performed with the ECL Western Blotting Analysis System (GE Healthcare, Broendby, Denmark).

Immunostaining for surface protein was performed by fixing RPMI-8226 in 4% formaldehyde for 20 minutes at room temperature (RT). After they were washed once in distilled water, cells were either smeared on a poly-L-lysine-coated glass slide or stained in

suspension. Cells were permeabilized with 0.1% (v/v) Triton X-100 for 10 minutes and the excess was washed twice with PBS. Samples were blocked with 1% (v/v) normal goat serum (NGS) for 90 minutes at RT. The anti-P2X7R-ec antibody was used at a concentration of 1.15 μg/mL diluted in NGS. Samples where the primary antibody was excluded in NGS were used as non-specific staining control. After they were incubated for 1 hour at RT, samples were washed 3 times in PBS. Alexa Fluor 488 goat anti-mouse IgG (5 μg/mL) was applied for 1 hour at RT in the dark and samples were washed 3 times in PBS and counterstained using Hoechst (1 μg/mL) for 20 minutes. After a final washing, samples on glass slides were mounted with ProLong Gold reagent for microscopy, and suspension was analyzed by flow cytometry.

7. *Measurement of cell viability and cell numbers*

An assessment of active metabolism, i.e., cleavage of tetrazolium salt into formazan, was used to measure cell viability. RPMI-8226 was seeded in duplicates at a density of 10^6 per mL in the presence of BzATP (0-300 μM). After 48 hours, cell proliferation reagent (WST-1, Roche Diagnostics) was added (10 %, v/v) directly to cell media and incubated for 1 hour at 37°C/5% CO_2. Absorbance of the formazan product was determined at 450 nm using a microplate reader. Wells containing the same dilution of WST-1 in cell medium (no cells) were used to determine the background absorbance. After the measurements, RPMI-8226 were lysed in 0.2% Triton-X in nuclease-free water (containing 20 mM Tris (pH 8.0-8.3); 1 mM $MgCl_2$) and frozen at −80°C. Lysates were thawed and dsDNA was measured with the Quant-iT PicoGreen dsDNA Assay Kit, according to the manufacturer's instructions, as a representation of cell numbers.

8. *Detection of apoptosis and necrosis*

Positive staining with annexin V and propidium iodide (PI) was used to determine the proportion of apoptotic (annexin V+/PI-) or necrotic (annexin V+/PI+) cells. RPMI-8226 were seeded in duplicates at a density of 10^6 per mL in the presence of 300 μM BzATP for chronic P2X7 receptor activation, 48 hours prior to assay. Cells were rinsed once with PBS (containing 2 mM EDTA) and stained with the Dead Cell Apoptosis Kit, according to the manufacturer's instructions. Stained cells were kept on ice and protected from light until analyzed. Using a FACSVerse (BD Biosciences), we excited the fluorochromes with lasers at 488 nm (filter 527/32 nm, green for annexin V and filter 586/42 nm, red for PI). Gating on the scatter dot plots was used to exclude cell debris and doublets and three groups of populations containing live (low levels of fluorescence), apoptotic (green fluorescence), and necrotic cells (both red and green fluorescence) were quantified. The assay was repeated on 4 different occasions and cells treated with Velcade (100nM) were used as a positive control of treatment. Unstained cells (no PI, no annexin V Alexa Fluor 488) were run on the instrument to set optimal voltage before collecting 10^5 events in each treatment group.

9. *Cell cycle progression*

RPMI-8226 were seeded in the presence of BzATP, as described in Section 8. At the indicated times, we labelled cells with 10 μM BrdU for 2 hours at 37°C/5% CO_2. BrdU labelling was

stopped by adding 70% ice-cold ethanol and centrifugation to remove excess BrdU. RPMI-8226 were fixed as an even suspension by dropwise addition of 70% ice-cold ethanol and vortexed followed by storage at 4°C overnight. On the day of staining, fixative was removed, and cellular DNA denatured in 2N HCl for 30 minutes. After neutralization of acid (50% v/v 0.1M borate buffer), nonspecific epitopes were blocked by incubation in PBS containing 0.0025% Tween 20 and 0.5% bovine serum albumin (PBS-T) for 1 hour at RT. Staining was performed on a rocking platform with Alexa Fluor 488 anti-BrdU for 1 hour at room temperature. After rinsing twice in PBS-T, cells were resuspended in PBS-T containing 5 μg/mL DAPI before being analyzed by flow cytometry. The fluorochromes were excited with lasers at 488 nm (Filter 527/32, for Alexa Fluor 488) and at 405 nm (Filter 448/45, for DAPI). Single cells were gated on a dot plot between forward and side scatter and the resulting population was analyzed for percentages in G0/G1, S, and G2/M phases. The assay was repeated on 3 different occasions and unlabeled cells were used as a negative control for BrdU staining.

10. Western blotting for caspase-3, and NF-κB

RPMI-8226 in lysis buffer was sonicated for 3 x 10 s and cellular debris was removed by centrifugation. Precleared lysates were measured for protein content using the Biorad DC protein assay and normalized to 20 μg total protein in 1 x lithium dodecyl sulfate (LDS) buffer prior to loading onto 4-20% gradient gels (Expedeon), being separated for 65 min at 180V and using 110 mA/gel. Proteins were electroblotted onto a 0.2 μm PVDF membrane (GE Healthcare) for 80 min at 150V and 350 mA. Prior to primary antibody incubation, the resulting membranes were blocked in 2% ECL Prime Blocking Agent (GE Healthcare) for 1 h at RT. Primary antibody incubation was ON on rotor at 4C in TBS-T + 2% ECL Blocking Agent + 0.01% NaAzide. After 2 x 5 min washes in TBS-T, the membranes were then incubated for 1 h on rotor at RT in secondary antibody solutions also containing TBS-T + 2% ECL Blocking Agent and NaAzide. After 5 x 5 min washes with TBS-T, the membranes were developed using ECL Select Western Blotting detection reagent for 5 min at RT. Image capture by LAS-4000 cooled the CCD camera. Primary antibodies used p- *NF-κB*, t- *NF-κB* -, and GAPDH, all from Cell Signaling (all raised in rabbits), used at 1:1.000, except GAPDH, which was used at 1:10.000. Secondary antibodies were goat-anti-mouse (Pierce) used at 1:40.000 and donkey-anti-rabbit (GE Healthcare) for all other primary antibodies, used at 1:40.000.

11. Quantitative PCR using TaqMan probes for IL-6, gp80, gp130

Total RNA was reverse-transcribed to cDNA, as described in Section 3. Inventoried Taqman assay probes with primers spanning across two exons (IL-6 Hs00174131_m1, IL6R/gp80 Hs01075664_m1, IL6ST/gp130 Hs00174360_m1) were combined with TaqMan 2x Universal PCR Master Mix according to the manufacturer's instructions. Each sample was run in triplicate and Ct ≥ 35 were not considered for analysis. Fold changes were calculated as 2ˆ(–delta delta CT), where deviation from 1 is considered an up- or downregulation.

12. Co- culture assay

A modified version of the Boyden chamber (Figure S1) using Corning HTS Transwell 96 well permeable supports (1.0 μm pore size, polyethylene membrane) was used to study cell interactions for 48 hours. To do this, RPMI-8226 (HMCL) was treated with 300μM BzATP (with or without 30-minute pretreatment with 1μM AZ11645373) for 90 minutes. After centrifugation to remove excess agonist (and antagonist), RPMI-8226 was added in suspension to the top well. Prior to assembly of the culture system, we grew osteoblasts and osteoclasts separately. A representative sample of osteoblasts and resorbing osteoclasts was tested with alkaline phosphatase staining or with toluidine blue stain [27], respectively, before being introduced into the co-culture system. The medium was a 1:1:1 v/v/v of HMCL, osteoblast, and osteoclast culture media and included ascorbic acid (50μg/mL), dexamethasone (10nM), hM-CSF (25ng/mL), and shRANKL (30ng/mL). The assembly was dismantled after 48 hours to assess 3 functional outputs, namely, RPMI-8226 viability (suspension used for WST-1 assay described in Section 7), osteoblast mineralization (transwell fixed and stained with 40 mM, pH 4.2 Alizarin Red) and resorption (dentine discs fixed and tartrate-resistant acid phosphatase stained).

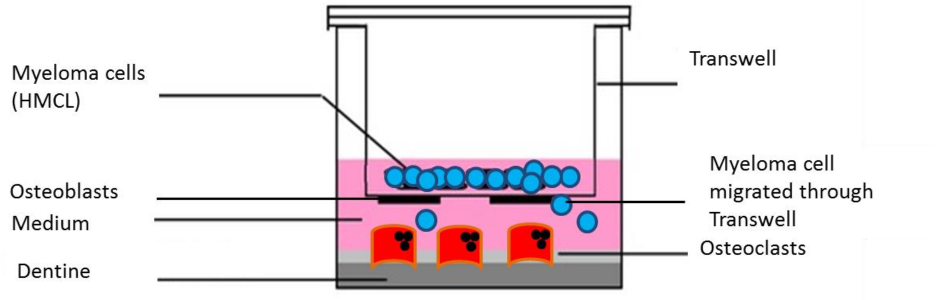

Figure S1. Setting for triple cell culture system. RPMI-8226 , treated with agonist and/or antagonist, was added in suspension to the top of transwell insert (pore size of 1 μm). Osteoblasts had been differentiating on the underside of the inserts, and dentine disk with resorbing osteoclasts was introduced to the bottom of the transwell.

13. Statistical Analysis

All quantitative data are expressed as a change of the relevant control group. Each treatment group was tested in replicate wells and mean values were calculated. Statistical analysis was performed on GraphPad PRISM version 7 using Student's *t*-test (Mann–Whitney test for non-parametric analysis, as indicated) or Kruskal–Wallis comparison across all treatment groups. All results, where appropriate, are presented as means ± SEM, and $p < 0.05$ was considered significant.
